# Identification of a new export signal that targets early subunits to the flagellar type III secretion export machinery

**DOI:** 10.1128/mbio.03067-23

**Published:** 2024-02-20

**Authors:** Owain J. Bryant, Gillian M. Fraser

**Affiliations:** 1 Department of Pathology, University of Cambridge, Tennis Court Road, Cambridge, United Kingdom; 2 Center for Structural Biology, Center for Cancer Research, National Cancer Institute, Frederick, Maryland, USA; University of Washington, Seattle, Washington, USA

**Keywords:** bacterial flagella biogenesis, type III secretion, protein export

## Abstract

Type III secretion systems (T3SSs) are essential for motility and virulence in many bacterial pathogens. Proteins destined for the flagellar T3SS contain at least two export signals in their N-terminal D_0_ domain. Here, we describe a third carboxy (C)-terminal signal in early flagellar subunits that facilitates subunit targeting to the export machinery. Mutational analysis identified critical residues within the flagellar hook subunit C-terminal export signal. The flagellar ATPase and cytoplasmic ring components were not required for this targeting, indicating that core export machinery components facilitate substrate targeting via the C-terminal export signal. More broadly, these results demonstrate that multiple distinct export signals within type III secretion substrates facilitate distinct export events at the T3SS export machinery. Our data establish key events in the export mechanism of type III secretion systems: targeting of subunits to and their sequential interactions with key components of the export machinery.

**IMPORTANCE:**

Many bacterial pathogens utilize T3SS to inject virulence proteins (effectors) into host cells or to assemble flagella on the bacterial cell surface. Bacterial flagella present a paradigm for how cells build and operate complex cell-surface “nanomachines.” Efficient subunit targeting from the bacterial cytosol to type III secretion systems is essential for rapid assembly and secretion by T3SSs. Subunits are thought to dock at the export machinery before being unfolded and translocated into the export channel. However, little is known about how subunits dock at the export machinery and the events that occur post docking. Here, we identified a new export signal within the C-termini of subunits that is essential for targeting of subunits to the type III export machinery. We show that this new export signal and previously identified export signals are recognized separately and sequentially, revealing a pathway for subunit transit through the type III export machinery in which sequential recognition events carry out different roles at major steps in the export pathway.

## INTRODUCTION

**Fig 1:**
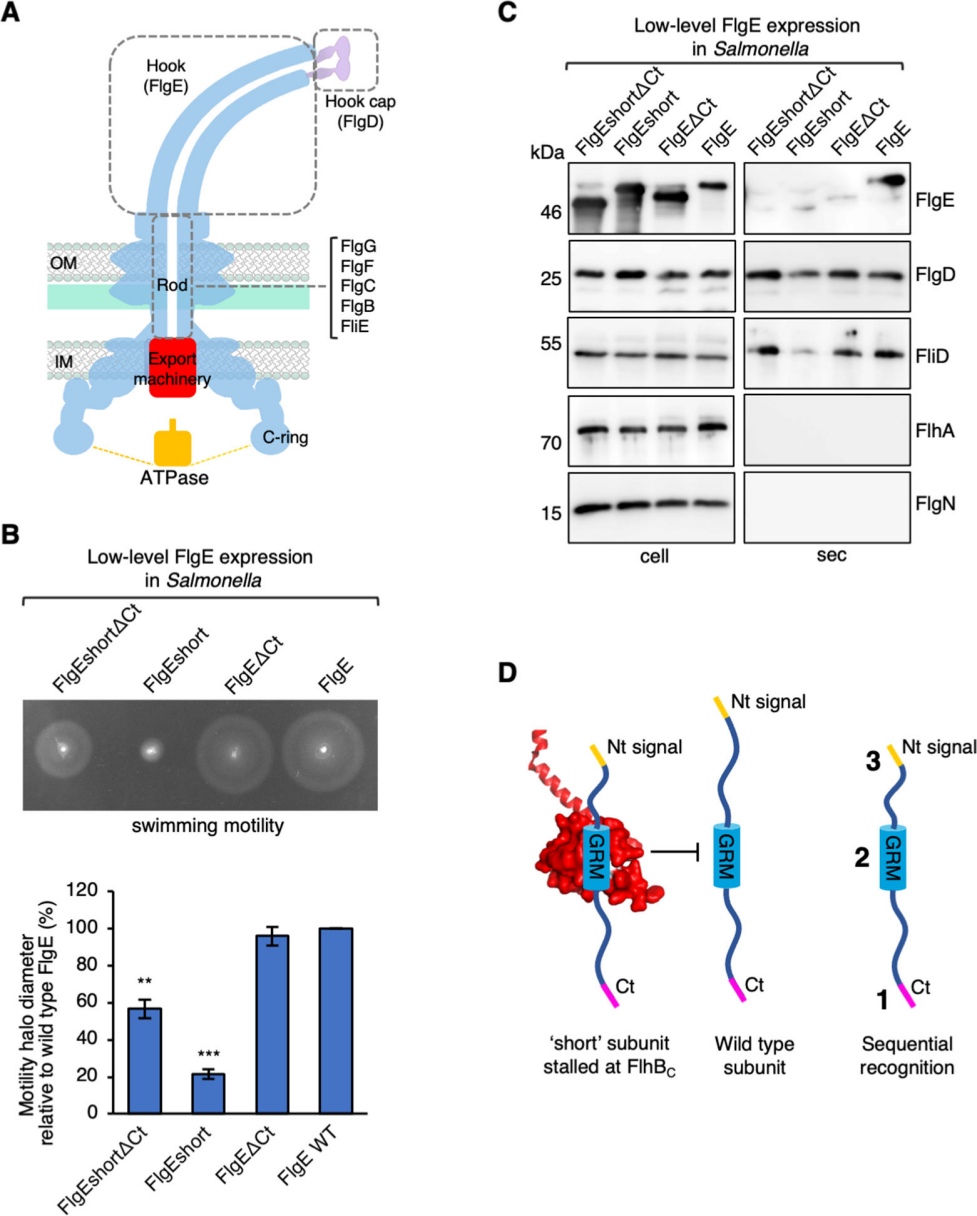
Effect on subunit export by expression of FlgEshort and variants. (**A)** A schematic of the bacterial flagellar basal body highlighting the location of the rod, hook, and hook cap structures (gray dashed boxes), the core components of the export machinery (red), the ATPase complex (yellow), and the cytoplasmic ring (C-ring). (**B)** Swimming motility of a wild-type (WT) *Salmonella* strain expressing plasmid-encoded wild-type FlgE containing an internal triple FLAG-tag (FlgE) or its variants (FlgEΔCt, FlgEΔshort, or FlgEshortΔCt). Motility was assessed in 0.25% soft-tryptone agar containing 100-µg/mL ampicillin and 50-µM isopropyl β-D-thiogalactoside and incubated for 4–6 h at 37°C. The diameter of motility halos was quantified and plotted as a percentage of motility halo diameter of FlgE wild type. The error bars represent the standard error of the mean calculated from three biological replicates. **P < 0.01, ***P < 0.001. (**C)** Whole cell (cell) and secreted (sec) proteins from late exponential-phase cultures were separated by SDS (15%)-PAGE and analyzed by immunoblotting with anti-FLAG (FlgE), anti-FlgD (hook cap subunit), anti-FliD (filament cap subunit), anti-FlhA (component of the export machinery), and anti-FlgN (export chaperone for FlgK and FlgL) polyclonal antisera. Apparent molecular weights are in kilodalton. Three biological replicates were performed. (**D)** A model depicting a “short” subunit (left) docked via its FlhB_C_ gate-recognition motif (GRM, blue) at the subunit binding pocket on FlhB_C_ [PDB: 3B0Z (16), red], preventing wild-type subunits (middle) from docking at FlhB_C_. A model depicting a typical early flagellar T3SS subunit (right) containing a N-terminal hydrophobic signal (yellow), a FlhB_C_ gate-recognition motif (GRM, blue), and a C-terminal export signal (magenta) which are recognized sequentially.

The bacterial flagellum is a large macromolecular nanomachine that enables bacteria to swim. The flagellum is assembled from thousands of subunits that are exported across the inner membrane by a specialized type III secretion export machinery located at the base of the flagellum (1, 2). The flagellar structures are assembled in a strict order with the basal body and export machinery being constructed first (3). Upon assembly of the export machinery, early flagellar subunits (FlgB, FlgC, FlgD, FlgE, FlgF, FlgG, FliE, FlgJ, and FliK) are exported and assemble to form the flagellar rod and hook substructures (Fig. 1A). After rod and hook substructure assembly has been completed, the late flagellar subunits (FlgK, FlgL, FliC, and FliD) are exported and assemble to form the more distal filament (1, 2). These subunits must be efficiently targeted from their site of synthesis at the ribosome to the type III secretion system (T3SS) export machinery (1, 4–7). This is achieved by a combination of targeting signals within the subunit RNA and/or polypeptide, and/or by targeting signals in chaperones that deliver subunits to the export machinery (8–15). Unlike late flagellar subunits, the early flagellar subunits are not chaperoned to the export machinery; therefore, all targeting signals are presumably contained within the subunit.

Early subunits dock at the FlhB_C_ component of the FlhAB-FliPQR export gate via a small hydrophobic motif termed the gate-recognition motif (GRM) located within the N-terminus (6, 7, 17, 18). We recently identified a second export signal at the extreme N-terminus of flagellar rod and hook subunits which triggers export gate opening (7). We showed that deletion of the gate-recognition signal (GRM) can overcome the dominant-negative overexpression phenotype of subunits deleted for the extreme N-terminal signal, indicating that the N-terminal export signal is recognized by the flagellar export machinery only after subunits have docked at FlhB_C_ via the GRM (7). The GRM and extreme N-terminal signals are utilized deep within export machinery, raising the possibility that other signals reside within early subunits for initial targeting to the export machinery, analogous to targeting of late export subunits by dedicated chaperones (12, 13, 19). We reasoned that we could screen for further export signals required for initial targeting of subunits to the export machinery by exploiting the fact that subunit variants defective in using the extreme N-terminal signal induce a dominant-negative overexpression phenotype which can be reversed by preventing the entry of subunits into the export pathway. We have previously shown that the C-termini of early flagellar subunits are required for efficient subunit export and proposed that early subunits docked at the FlhB_C_ are captured via the C-terminus of the preceding subunit already in the export channel (17). Here we provide data indicating an alternative role for early flagellar subunit C-termini in subunit export and show that early subunit C-termini contain an export signal required for initial targeting of subunits to the export machinery. The data establish key events in the export mechanism of the flagellar type III secretion systems: targeting of subunits and their sequential interactions with key components of the export machinery.

## RESULTS

### Identification of an export signal in the C-terminus of rod and hook subunits

Subunit variants defective in using the extreme N-terminus stall at the FlhB component of the export gate and are dominant-negative for export and motility (7). Deletion of the FlhB GRM reverses the dominant-negative phenotype by preventing subunits from stalling at FlhB (7). We reasoned that subunit variants defective in entering the export pathway could similarly reverse the dominant-negative phenotype and be used as a screen to identify export signals that are used in the export pathway before the extreme N-terminal export signal. We previously showed that deletion of the C-terminus of FlgE attenuates subunit export (17). To determine whether the C-terminus of FlgE is used prior to the extreme N-terminal export signal of FlgE, we assessed whether deletion of the FlgE C-terminus reverses the dominant-negative phenotype of an FlgE variant defective in using the extreme N-terminal export signal (FlgEshort, Table 1). To test this, recombinant expression vectors encoding FlgE variants (FlgEshort∆Ct, FlgEshort, and FlgE∆Ct) and wild-type (WT) FlgE were introduced into a wild-type *Salmonella* strain and expressed in *trans* at the same level as the endogenous FlgE subunit (Fig. 1B; Fig.S1). Motility and subunit export assays revealed that the FlgEshort, ∆Ct variant did not induce a dominant-negative phenotype, unlike the FlgEshort variant, which did. This indicates that removal of the FlgE C-terminus reverses the dominant- negative phenotype of FlgEshort, indicating that the FlgE C-terminus is used before the extreme N-terminal signal on FlgE is used (Fig. 1B and C). The same export phenotypes were observed in a non-assembling flagella *Salmonella* strain (Fig. S2A). In contrast, FlgE∆Ct did not inhibit motility or subunit export compared to wild-type FlgE when expressed at close to endogenous levels. Unexpectedly, overexpression of full-length FlgE attenuated *Salmonella* motility and flagellar subunit export to a greater extent than overexpression of FlgEΔCt (Fig. 3A and B). This indicates that the presence of the C-terminus rather than its absence results in a dominant-negative overexpression phenotype.

**TABLE 1:** Subunit variant nomenclature

Subunit variant	Amino acid deletion
FlgEshort	FlgEΔ9–32
FlgEΔCt	FlgEΔ360–403
FlgEshortΔCt	FlgEΔ9–32, Δ360–403
FlgEshort + linker	FlgE1-8, 4× (Gly-Ser-Thr-Asn-Ala-Ser), 33–403 aa
FlgEΔGRM	FlgEΔ39–43
FlgGΔCt	FlgGΔ218–260
FlgCΔCt	FlgCΔ91–134
FlgDΔCt	FlgDΔ191–232

We supposed that if the FlgE C-terminus contains an export signal required for subunit targeting to the export machinery, then increasing the expression of FlgEΔCt may rescue its export defect, as has been observed for late flagellar export subunits deleted for their targeting signals (20). To test this, we assessed the secretion capability of a *Salmonella* Δ*flgD* strain (deficient in hook assembly) expressing wild-type FlgE or FlgEΔCt at endogenous levels or at elevated expression levels (Fig. S3A). We found that cells expressing higher levels of FlgEΔCt restored the levels of FlgEΔCt in culture supernatants to that of wild-type FlgE (Fig. S3A). These observations suggest that the FlgE C-terminus is required for efficient FlgE export and that increasing the levels of FlgEΔCt restores the export defect (Fig. S3A).

**Fig 2:**
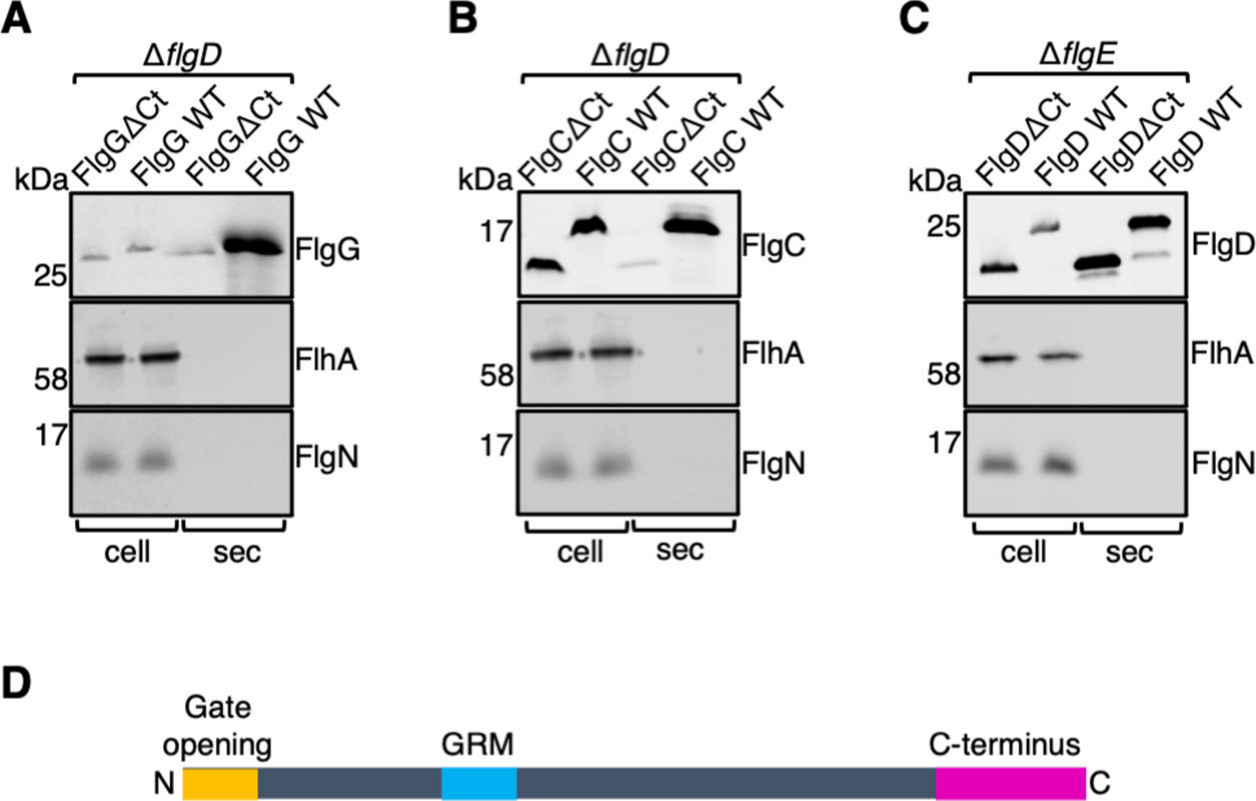
A subset of early flagellar subunits contains an export signal within the C-terminus. (**A)** Secretion analysis of a *Salmonella* Δ*flgD* strain transformed with recombinant pTrc99a plasmids carrying full-length FlgC (FlgC WT) or its variant lacking residues 91–134 (FlgCΔCt). FlgC subunits were engineered to contain an internal 3× FLAG tag for immunodetection. Cultures were grown in LB containing 100-µg/mL ampicillin and 50-µM IPTG. Whole cell (cell) and secreted (sec) proteins from late exponential-phase cultures were separated by SDS-PAGE and immunoblotted with polyclonal anti-FLAG (FlgC), anti-FlhA, or anti-FlgN antisera. Apparent molecular weights are in kilodalton. Three biological replicates were performed. (**B)** Secretion analysis of a *Salmonella* Δ*flgD* strain transformed with recombinant pTrc99a plasmids carrying full-length FlgG (FlgG WT) or its variant lacking residues 218–260 (FlgGΔCt). FlgG subunits were engineered to contain an internal 3× FLAG tag for immunodetection. Cultures were grown in LB containing 100-µg/mL ampicillin and 50-µM IPTG. Cell and sec proteins from late exponential-phase cultures were separated by SDS-PAGE and immunoblotted with polyclonal anti-FLAG (FlgG), anti-FlhA, or anti-FlgN antisera. Apparent molecular weights are in kilodalton. Three biological replicates were performed. (**C)** Secretion analysis of a *Salmonella* Δ*flgE* strain transformed with recombinant pTrc99a plasmids carrying full-length FlgD (FlgD WT) or its variant lacking residues 191–232 (FlgDΔCt). FlgD subunits were engineered to contain an internal 3× FLAG tag for immunodetection. Cultures were grown in LB containing 100-µg/mL ampicillin and 50-µM IPTG. Cell and sec proteins from late exponential-phase cultures were separated by SDS-PAGE and immunoblotted with polyclonal anti-FLAG (FlgD), anti-FlhA, or anti-FlgN antisera. Apparent molecular weights are in kilodalton. Three biological replicates were performed. (**D)** Schematic representation of a FlgE subunit containing an N-terminal hydrophobic signal (orange, labeled as gate-opening), an FlhB_C_ gate-recognition motif (GRM) (blue) and C-terminal signal (magenta, labeled as C-terminus).

To investigate whether the C-termini of other early flagellar subunits (subunits that assemble to form the rod and hook substructures) are required for their efficient export, we constructed subunit variants of the flagellar hook cap (FlgD), minor rod (FlgC), and major rod (FlgG) that lacked their C-termini. These C-terminal deletion variants and their corresponding wild-type subunits were expressed in *Salmonella* non-assembling flagella strains deficient in hook assembly [Δ*flgE* (for FlgD) or Δ*flgD* (for FlgC and FlgG)]. All subunit variants were stably expressed; however, export of both the FlgCΔCt and FlgGΔCt variants was attenuated compared to their corresponding wild-type subunits (Fig. 2A and B). In contrast, no export defect was observed for the FlgDΔCt variant (Fig. 2). These observations suggest that FlgD does not contain a C-terminal export signal. We supposed that if FlgD lacks this C-terminal export signal, then overexpression of the FlgD C-terminal deletion variant (FlgDΔCt) would not induce a dominant-negative overexpression phenotype compared to wild-type FlgD (as was seen for overexpression of FlgE variants containing the C-terminal export signal). The dominant-negative overexpression phenotypes of wild-type FlgD and FlgDΔCt were examined by assessing the swimming motility of the *Salmonella* Δ*recA* strain expressing the FlgD variants (Fig. S3B). We found that there was no difference in motility of cells expressing wild-type FlgD or FlgDΔCt (Fig. S3B), consistent with FlgD not containing a C-terminal export signal.

**Fig 3:**
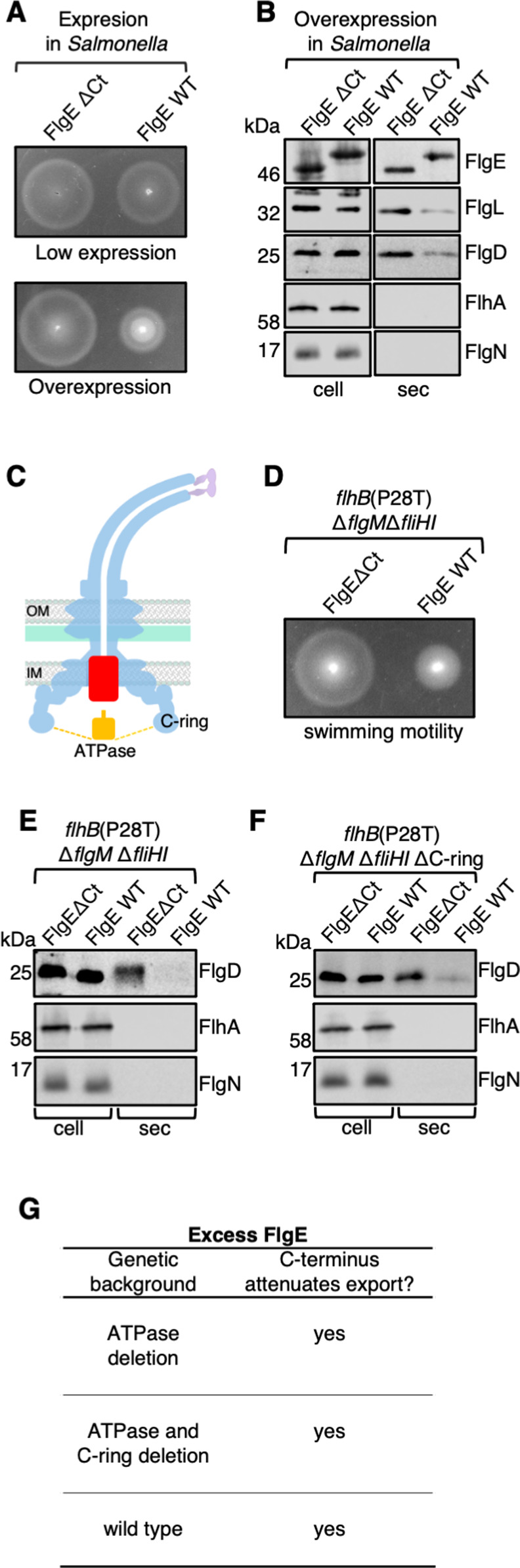
FlgE exerts a dominant-negative overexpression phenotype. (**A)** Swimming motility assays of a *Salmonella* Δ*recA* strain transformed with recombinant pTrc99a plasmids carrying full-length FlgE (FlgE WT) or its variant lacking residues 360–403 (FlgEΔCt). FlgE subunits were engineered to contain an internal 3× FLAG tag for immunodetection. Motility was assessed in 0.3% soft-tryptone agar containing 100-µg/mL ampicillin and 50-µM isopropyl β-D-thiogalactoside (IPTG) (low expression) or 1-mM IPTG (overexpression) and incubated for 4–6 h at 37°C. Three biological replicates were performed. (**B) **Secretion analysis of a *Salmonella* Δ*recA* strain transformed with recombinant pTrc99a plasmids carrying full-length FlgE (FlgE WT) or its variant lacking residues 360–403 (FlgEΔCt). FlgE subunits were engineered to contain an internal 3× FLAG tag for immunodetection. Cultures were grown in Luria-Bertani (LB) containing 100-µg/mL ampicillin and 1-mM IPTG (overexpression). Whole cell (cell) and secreted (sec) proteins from late exponential-phase cultures were separated by SDS-PAGE and immunoblotted with polyclonal anti-FLAG (FlgE), anti-FlgL, anti-FlgD, anti-FlhA, or anti-FlgN antisera. Apparent molecular weights are in kilodalton. Three biological replicates were performed. (**C)** A schematic of the bacterial flagellum highlighting the location of the core components of the export machinery (red), the ATPase complex (yellow), and the C-ring. (**D)** Swimming motility assays of a *Salmonella* strain [labeled *flhB*(P28T) Δ*fliHI*, and Δ*flgM*] deleted for the genes encoding the ATPase components FliH and FliI, and the anti-sigma factor (FlgM) in combination with the suppressor mutation in the *flhB* gene (P_28_T). This strain was transformed with recombinant pTrc99a plasmids carrying an FlgE variant containing an internal 3× FLAG tag (labeled FlgE WT) or its variant deleted for residues 360–403 (FlgEΔCt). Motility was assessed in 0.3% soft-tryptone agar containing 100-µg/mL ampicillin and 1-mM IPTG (overexpression) and incubated for 4–6 h at 37°C. Three biological replicates were performed. (**E)** Secretion assays of the above strains. Cultures were grown in LB containing 100-µg/mL ampicillin and 1-mM IPTG (overexpression). Cell and sec proteins from late exponential-phase cultures were separated by SDS-PAGE and immunoblotted with polyclonal anti-FlgD, anti-FlhA, or anti-FlgN antisera. Apparent molecular weights are in kilodalton. Three biological replicates were performed. (**F)** Secretion analysis of a *Salmonella* strain [*flhB*(P28T), Δ*fliHI*, and ΔC-ring] deleted for the genes encoding the ATPase components FliH and FliI, the C-ring components FliM and FliN, and the anti-sigma factor (FlgM) in combination with the suppressor mutation in the *flhB* gene (P_28_T). This strain was transformed with recombinant pTrc99a plasmids carrying an FlgE variant containing an internal 3× FLAG tag (labeled FlgE WT) or its variant deleted for residues 360–403 (FlgEΔCt). Cultures were grown in LB containing 100-µg/mL ampicillin and 1-mM IPTG (overexpression). Cell and sec proteins from late exponential-phase cultures were separated by SDS-PAGE and immunoblotted with polyclonal anti-FlgD, anti-FlhA, or anti-FlgN antisera. Apparent molecular weights are in kilodalton. Three biological replicates were performed. (**G)** Summary of the effect of FlgE overexpression on the attenuation of subunits other than FlgE in different genetic backgrounds. Overexpression of FlgE attenuates the export of subunits other than FlgE in the absence of the ATPase and C-ring components and is dependent on the presence of the C-terminal export signal. These data indicate that the FlgE C-terminus does not act at the ATPase or C-ring but at one of the core components of the export machinery.

### Attenuation of motility and flagellar subunit export associated with FlgE overexpression does not require the flagellar export ATPase complex or the cytoplasmic ring

We reasoned that the C-terminus of FlgE may bind and either stall at or sequester one of the components of the export machinery, and that attenuation of motility and subunit export upon overexpression of FlgE would be lost in an export competent strain deleted for the C-terminus binding partner (Fig. 3A through C). To test whether the flagellar ATPase complex is a binding partner, we overexpressed FlgEΔCt and wild-type FlgE in a motile strain of *Salmonella* that contained deletions in the genes that encode the FliH and FliI components of the ATPase in combination with mutations that bypass the need for the ATPase complex (*flhB*P_28_T*-*Δ*fliHI*-Δ*flgM*)^21^ (Fig. S4). Motility and export assays revealed that overexpression of wild-type FlgE attenuated subunit export and cell motility and that deletion of the FlgE C-terminus suppressed this overexpression phenotype (Fig. 3D and E), indicating that overexpressed FlgE does not bind and sequester the flagellar ATPase complex.

To test whether the flagellar cytoplasmic ring (C-ring) is a possible binding partner for the FlgE C-terminus, we constructed a strain deleted for genes encoding the C-ring (*fliM* and *fliN*) and deleted for the genes encoding the FliH and FliI components of the ATPase complex, in combination with mutations that bypass the need for the ATPase complex (*flhB*P28T*-*Δ*fliHI-*Δ*fliMN-*Δ*flgM*). This strain is non-motile; deletion of the C-ring prevents directional rotation of the bacterial flagellum, as well as prevents localization of the flagellar ATPase complex at the export machinery (22). Strains deleted for genes encoding the C-ring are therefore non-motile; however, a P28T mutation in *flhB* restores protein export, and subunits are still exported into culture supernatants (Fig. 3F). We overexpressed FlgEΔCt and wild-type FlgE in this strain and found that wild-type FlgE attenuated export of subunits into culture supernatants, whereas overexpression of FlgEΔCt did not, suggesting that deletion of the FlgE C-terminus suppresses the overexpression phenotype (Fig. 3F). These results indicate that the FlgE C-terminus does not bind the flagellar ATPase or the C-ring (Fig. 3G). This leaves only the cytoplasmic domains of the core components of the export machinery, FlhA and FlhB, as potential binding partners for the FlgE C-terminus.

### Mutations within the FlgE hook subunit C-terminus attenuate export

**Fig 4:**
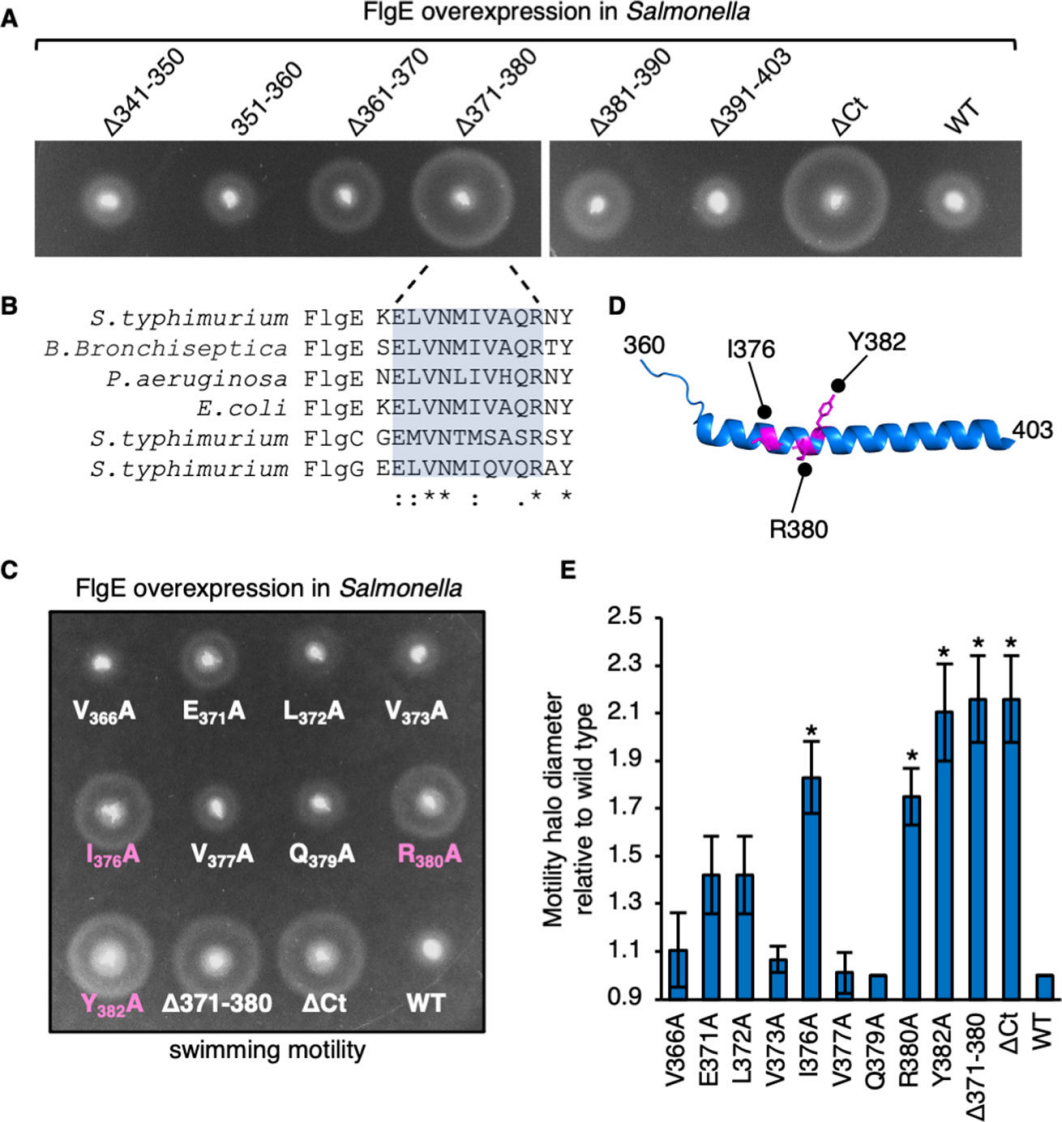
. Mutational analysis of the C-terminal export signal identifies critical residues within FlgE. (**A)** Swimming motility assays of a *Salmonella* Δ*recA* strain transformed with recombinant pTrc99a plasmids carrying full-length FlgE (labeled as WT) or its variants: Δ341–350, Δ351–360, Δ361–370, Δ371–380, Δ381–390, Δ391–403, or ΔCt. FlgE subunits were engineered to contain an internal 3× FLAG tag for immunodetection. Motility was assessed in 0.3% soft-tryptone agar containing 100-µg/mL ampicillin and 1-mM IPTG (overexpression) and incubated for 4–6 h at 37°C. Three biological replicates were performed. (**B)** Alignment of *S. typhimurium* FlgE sequence 370–382 with homologous regions in FlgE subunits from other bacterial species and with *S.typhimurium* FlgC and FlgG. (**C)** Swimming motility assays of a *Salmonella* Δ*recA* strain transformed with recombinant pTrc99a plasmids carrying full-length FlgE (labeled as WT) or its variants: V_366_A, E_371_A, L_372_A, V_373_A, I_376_A, V_377_A, Q_379_A, R_380_A, Y_382_A, Δ371–380, and ΔCt. FlgE subunits were engineered to contain an internal 3× FLAG tag for immunodetection. Motility was assessed in 0.3% soft-tryptone agar containing 100-µg/mL ampicillin and 1-mM IPTG (overexpression) and incubated for 4–6 h at 37°C. FlgE point mutation variants that reversed the dominant-negative overexpression phenotype are colored in magenta. Three biological replicates were performed. (**D)** Residues (magenta) required for the dominant-negative overexpression phenotype of FlgE mapped onto the C-terminal region (360-403) of FlgE (PDB: 6JZT (23). (**E)** The diameters of motility halos in panel **D** were quantified and plotted as a percentage of motility halo diameter for overexpression of wild-type FlgE. The error bars represent the standard error of the mean calculated from three biological replicates. *P < 0.05.

To identify specific sequences in the C-terminus of FlgE required for efficient export, internal 10-residue scanning deletions were made within the C-terminal 63-residue region, apart from FlgEΔ391–403, which removes the final 13 residues (Fig. 4A; Fig. S3). We utilized the overexpression phenotype of FlgE to screen for variants that had lost this phenotype, suggesting a loss of interaction between the subunit C-terminus and the export machinery. FlgEΔCt, wild-type FlgE, and its variants were overexpressed in a *Salmonella* Δ*recA* strain, and their swimming motility in soft-tryptone agar was assessed (Fig. 4A). The screen identified a single FlgE variant (FlgEΔ371–380) that suppressed the overexpression phenotype to the same extent as for the FlgEΔCt variant (Fig. 4A). This indicates that the export signal within the FlgE C-terminus is localized within residues 371–380. Residues 371–380 contain three fully conserved residues (V373, N374, and R380) and four partially conserved residues (E371, L372, II376, and Q379; Fig. 4B; Fig. S5 and S6). An alanine scanning mutagenesis approach was used to identify FlgE residues required to bring about the overexpression phenotype (Fig. 4C). A total of nine residues were substituted with alanine, seven of which were located within the 371–380 region (E371, L372, V373, N374, I376, Q379, and R380), and two fully conserved residues were located adjacent to the 371–380 region (V366 and Y382). We again utilized the dominant-negative overexpression phenotype of FlgE to screen for mutants that had lost this phenotype, suggesting a loss of subunit stalling at the export machinery (Fig. 4C through E; Fig. S6B). We found that the FlgE- I_376_A, FlgE-R_380_A, and FlgE-Y_382_A variants suppressed the dominant-negative overexpression motility phenotype of FlgE, indicating that these residues constitute part of the C-terminal export signal (Fig. 4C through E).

**Fig 5:**
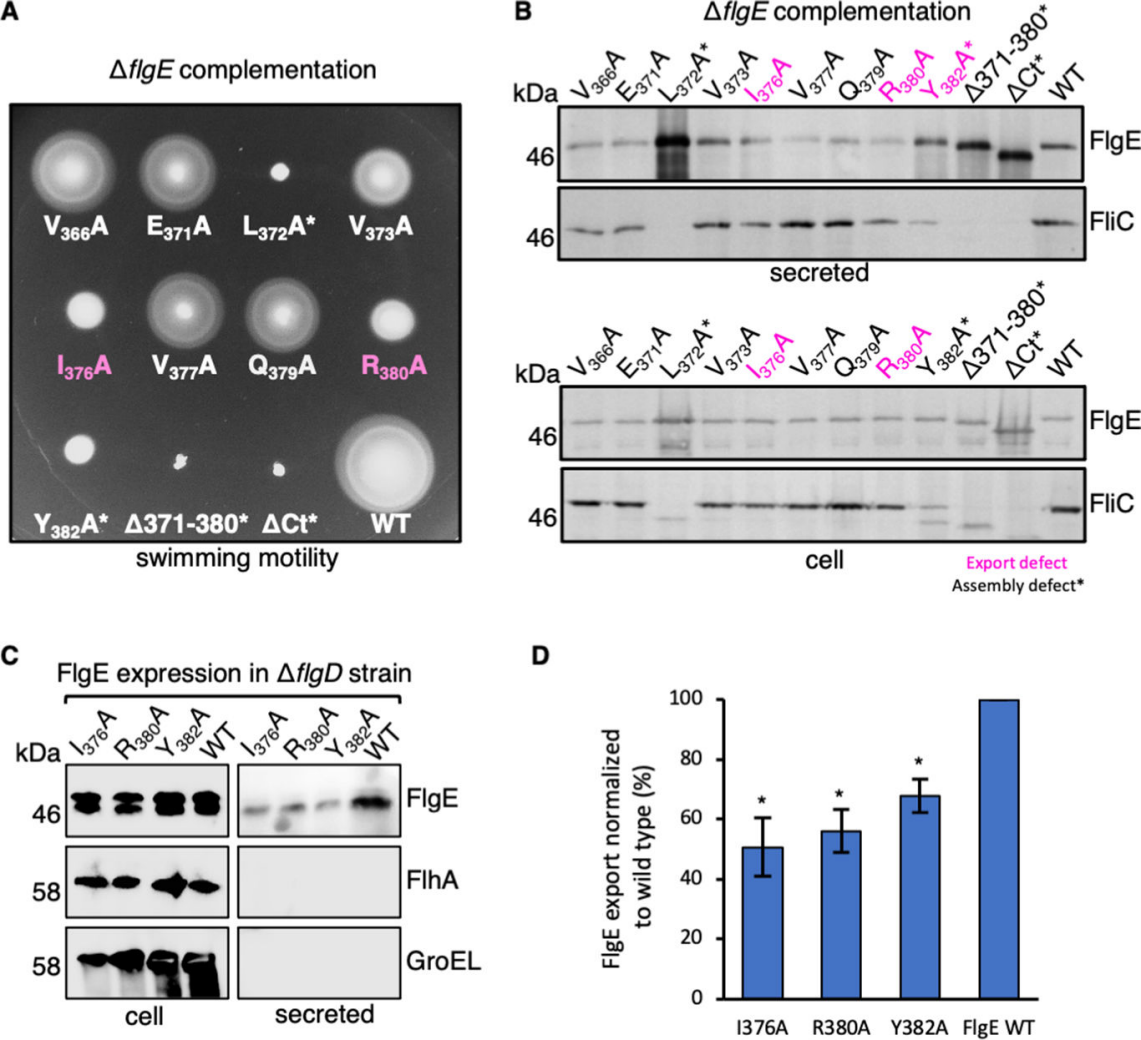
The FlgE C-terminus contains a targeting signal required for efficient subunit export. (**A)** Swimming motility assays of a *Salmonella* Δ*flgE* strain transformed with recombinant pTrc99a plasmids carrying wild-type FlgE (labeled as WT) or its variants: V_366_A, E_371_A, L_372_A, V_373_A, I_376_A, V_377_A, Q_379_A, R_380_A, Y_382_A, Δ371–380, and ΔCt. FlgE subunits were engineered to contain an internal 3× FLAG tag for immunodetection. Motility was assessed in 0.3% soft-tryptone agar containing 100-µg/mL ampicillin and 50-µM IPTG (endogenous expression) and incubated for 4–6 h at 37°C. FlgE point mutation variants that reversed the dominant-negative overexpression phenotype are colored in magenta. Three biological replicates were performed. (**B)** Secretion assays of the above strains. Cultures were grown in LB containing 100-µg/mL ampicillin and 50-µM IPTG. Secreted proteins (secreted, top panels) and whole cell proteins (cell, bottom panels) from late exponential-phase cultures were separated by SDS-PAGE and immunoblotted with monoclonal anti-FLAG (FlgE)lgE or polyclonal anti-FliC sera. Apparent molecular weights are in kilodalton. FlgE point mutation variants that reversed the dominant-negative overexpression phenotype are colored in magenta. Three biological replicates were performed. (**C)** Secretion assays of a *Salmonella* Δ*flgDE* strain (a non-assembling strain that can export FlgE but not assemble FlgE into the hook substructure) transformed with recombinant pTrc99a plasmids carrying wild-type FlgE (labeled as WT) or its variants: I_376_A, R_380_A, or Y_382_A. Cultures were grown in LB containing 100-µg/mL ampicillin and 50-µM IPTG. Secreted proteins (secreted, top panels) and whole cell proteins (cell, bottom panels) from late-exponential-phase cultures were separated by SDS-PAGE and immunoblotted with monoclonal anti-FLAG (FlgE), polyclonal anti-FlhA sera, or polyclonal GroEL sera. Apparent molecular weights are in kilodalton. Motility was assessed in 0.3% soft-tryptone agar containing 100-µg/mL ampicillin and 50-µM IPTG (low expression, top panel) or 100-µM IPTG (higher expression, bottom panel) and incubated for 4–6 h at 37°C. Three biological replicates were performed. (**D**) The relative secretion levels from panel C were quantified and plotted as a percentage of export of wild-type FlgE. The error bars represent the standard error of the mean calculated from three biological replicates. *P < 0.05.

The loss of the FlgE overexpression phenotype by the FlgE-I_376_A, FlgE-R_380_A, and FlgE-Y_382_A variants indicates that these mutants, like FlgEΔCt, have lost subunit targeting activity and would be exported less efficiently than wild-type FlgE, less efficient FlgE export would result in a slower rate of hook assembly. Completion of hook assembly must occur before filament assembly can begin, therefore delaying completion of hook assembly, and therefore flagellar filament assembly would attenuate swimming motility. To test this, FlgEΔCt, wild-type FlgE, and its variants (V_366_A, E_371_A, L_372_A, V_373_A, N_374_A, I_376_A, Q_379_A, R_380_A, and Y_382_A) were expressed at endogenous levels in a *Salmonella* Δ*flgE* strain (Fig. 5A and B). Swimming motility of cells in soft-tryptone agar revealed that four FlgE mutants (L_372_A, I_376_A, R_380_A, and Y_382_A) were less motile than wild type (Fig. 5A).

To test whether the FlgE-I_376_A, FlgE-R_380_A, and FlgE-Y_382_A variants were exported less efficiently into culture supernatants (consistent with a targeting defect), plasmids encoding the FlgE variants were transformed into a ∆*flgE* strain, and the FlgE variants were expressed at endogenous levels by induction with 50-µM isopropyl β-D-thiogalactoside (IPTG) (Fig. 5B). Levels of FlgE-L_372_A, FlgE∆371–380, and FlgE∆Ct in culture supernatants were greater than for wild type, consistent with these FlgE variants being deficient in hook assembly. Furthermore, whole cell and supernatant levels of FliC by these strains were attenuated, consistent with deficient hook assembly (and therefore a loss of the substrate specificity switching that follows completion of hook assembly) (Fig. 5B). Secretion defects by the other FlgE variants were subtle but indicate that hook assembly is not disrupted. These observations suggest that both FlgE-I_376_A and FlgE-R_380_A have reduced subunit targeting activity but retain the ability to assemble flagellar hooks.

To further confirm that the three FlgE variants identified in our above screen (FlgE-I_376_A, FlgE-R_380_A, and FlgE-Y_382_A) display reduced export efficiency, we expressed these variants (FlgE-I_376_A, FlgE-R_380_A, and FlgE-Y_382_A) in a non-assembling flagellar strain (∆*flgD*) that can export protein but not assemble the flagellar hood and more distal structures. In this way, we can uncouple the effects of FlgE mutations on flagellar protein export from flagellar assembly. This confirmed that FlgE-I_376_A, FlgE-R_380_A, and FlgE-Y_382_A are exported less efficiently into culture supernatants (Fig. 5C).

We screened for motile suppressors with improved swimming motility from the ∆*flgE* strain expressing FlgE-I_376_A or FlgE-R_380_A. Sequencing of these suppressors revealed that all suppressors contained second site mutations that mapped to the LacI repressor binding site of the heterologous *flgE* promoter region in pTrc99a, indicating that increasing the expression level compensates for the loss of subunit targeting to the export machinery and likely increases the chance of subunits reaching the export machinery to subsequently bind the FlhB_C_ (Fig. S6C). These data suggest that increasing the expression level of FlgE variants lacking the C-terminal export signal suppresses the export defect, consistent with our previous data showing overexpression of FlgE∆Ct restored export into culture supernatants (Fig. S3A).

### Attenuation of motility and subunit export by overexpression of subunit variants lacking export signals

**Fig 6:**
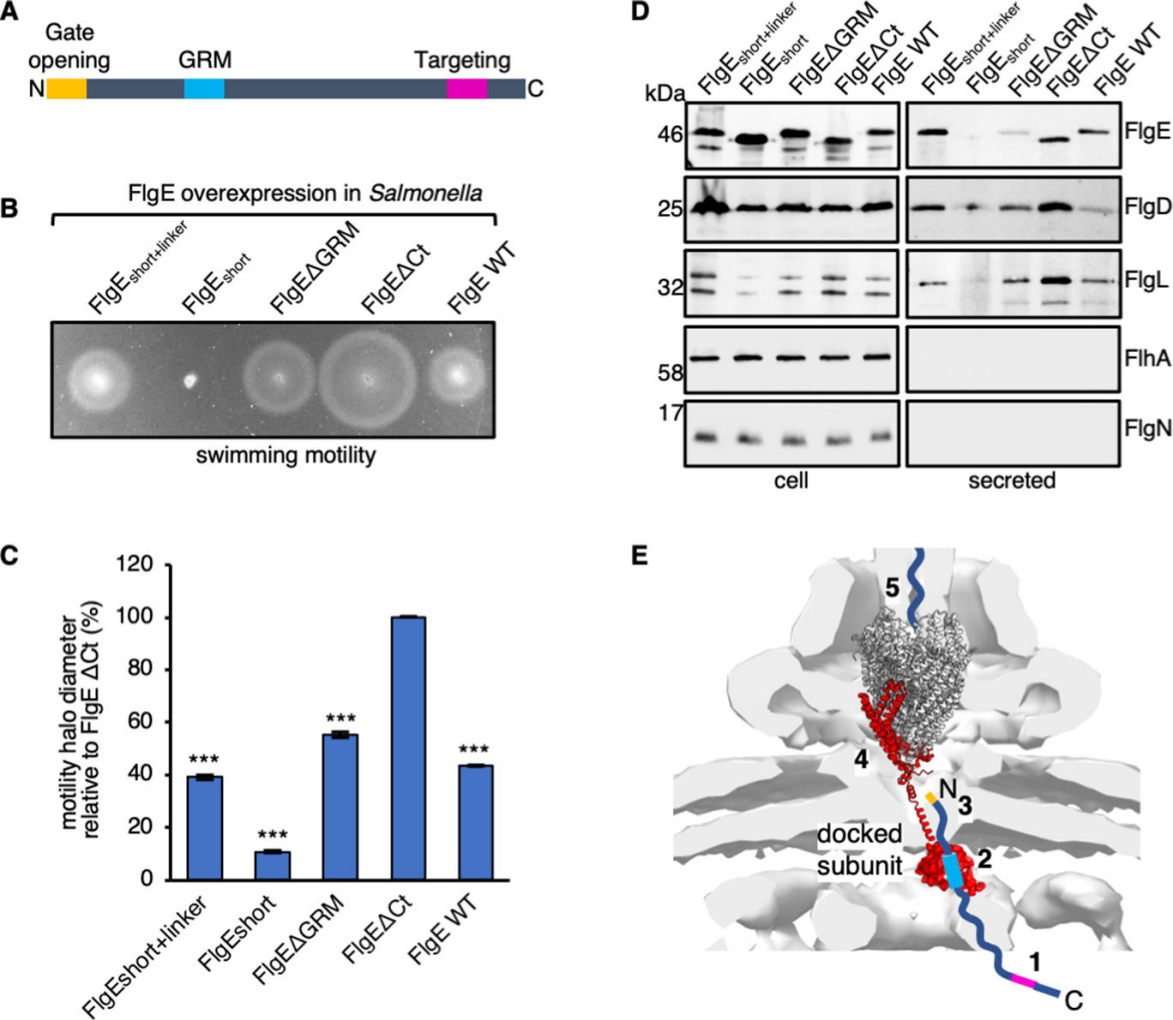
Overexpression phenotypes of FlgE_short_, FlgEΔGRM, FlgEΔCt, and their variants. (**A)** A schematic representation of an early flagellar subunit, highlighting the gate-opening signal (red), the gate-recognition motif (green), and the C-terminal targeting signal (magenta). (**B)** Swimming motility assays of a *Salmonella* Δ*recA* strain transformed with recombinant pTrc99a plasmids carrying full-length FlgE (labeled as FlgE EWT) or its variants: FlgE_short+linker_, FlgE_short_, FlgEΔGRM, and FlgEΔCt. FlgE subunits were engineered to contain an internal 3× FLAG tag for immunodetection. Motility was assessed in 0.3% soft-tryptone agar containing 100-µg/mL ampicillin and 1-mM IPTG (overexpression) and incubated for 4–6 h at 37°C. Three biological replicates were performed. (**C)** The diameter of motility halos in panel **B** were quantified and plotted as a percentage of motility halo diameter for overexpression of FlgEΔCt. The error bars represent the standard error of the mean calculated from three biological replicates. ***P < 0.001. (**D)** Secretion assays of the above strains. Cultures were grown in LB containing 100-µg/mL ampicillin and 1-mM IPTG. Whole cell proteins (cell) and secreted proteins (secreted) from late-exponential-phase cultures were separated by SDS-PAGE and immunoblotted with monoclonal anti-FlgE or polyclonal anti-FlgD, anti-FlgL, anti-FlhA, or anti-FlgN sera. Apparent molecular weights are in kilodalton. Three biological replicates were performed. (**E)** Model for the sequence of binding events between early subunits and the export machinery. Early flagellar subunits are targeted to the export machinery via their C-terminal export signal (magenta) (1). Subunits subsequently dock at the FlhB_C_ gate via their gate-recognition motif (GRM, blue) (2) positioning the N-terminal non-polar signal (yellow) (3) to trigger opening of the export gate complex (gray) (4). Subunits are subsequently translocated into the export channel at the center of flagellum (5), where they transit to the filament tip and refold into the nascent structure.

The accruing data indicate that the C-terminal export signal is used to target subunits to the export machinery before the subunit GRM and extreme N-terminal signals are recognized. We wanted to assess the severity of overexpression of FlgE subunit variants lacking all the so far identified FlgE export signals (N-terminal, GRM, or C-terminal signals) on motility and subunit export of a *Salmonella* strain that normally exhibits wild-type motility. To test this, we transformed a *Salmonella* ∆*recA* strain with pTrc99a encoding FlgE variants deleted for or defective in utilization of one of their three export signals: FlgE∆9–32 (or FlgEshort), FlgE∆GRM, or FlgE∆Ct (Fig. 6A). As a control, we used wild-type FlgE and a FlgE variant whereby amino acids 9–32 were replaced with four tandem repeats 4× (GSTNAS) to restore the length of the N-terminus (FlgE_short+linker_). Wild-type FlgE and its variants were overexpressed by induction with 1-mM IPTG. Motility and export assays revealed that the FlgE∆Ct, FlgE∆GRM, and FlgEshort variants displayed progressively more severe negative effects on motility and export. The same export phenotypes were observed when this experiment was performed with a non-assembling flagella strain (Fig. S7). Interestingly, the severity of these phenotypes correlates with the sequence in which the export signals are utilized by the export machinery, consistent with subunit variants that stall at later stages of the export cycle, inducing a more severe export defect.

## DISCUSSION

T3SS substrates that form the flagellar rod and hook or the injectisome inner rod and needle contain an FlhB GRM that docks them at the FlhB/SctU component of the export gate (7, 17). A second signal within the extreme N-terminus is recognized by the export machinery only after subunits have docked at FlhB_C_ via the GRM (7). Here, we show that the C-termini of a subset of early flagellar subunits contain an export signal that is recognized before the extreme N-terminal export signal and facilitates subunit targeting to the export machinery.

We have previously shown that export of a FlgE subunit variant lacking its C-terminal helix was attenuated (17). It was proposed that early subunits docked at FlhB_C_ are captured via the C-terminus of the preceding subunit already in the export channel and that these subunit capture events could result in a multi-subunit chain that spans from the FlhB_C_ export gate to the filament tip (1, 17). Evidence of this was obtained by *in vitro* capture assays, whereby subunits docked at FlhB_C_ could be captured by the C-terminus of other early subunits. Subunit variants lacking their C-termini would be captured from FlhB_C_ but unable to capture newly docked subunits, resulting in subunit stalling at FlhB_C_ (1, 17). This would result in a dominant-negative phenotype, whereby subunits would stall and not be captured from FlhB_C_, preventing other subunits from docking at FlhB_C_ and being exported. Here, we show that an FlgE hook subunit variant lacking its C-terminus does not display a dominant-negative phenotype. Instead, overexpression of wild-type FlgE (which contains the C-terminal region) attenuated subunit export and motility to a greater degree than FlgE∆Ct (Fig. 3A and B). This suggests that early flagellar subunits are not captured from FlhB_C_ by the C-termini of FlgE subunits as previously thought (17). We found that increasing the expression level of FlgE∆Ct restored export into culture supernatants to that of wild-type FlgE, indicating that the C-terminus of FlgE is not required for subunit capture from FlhB_C_ and may instead contain an alternative export signal (Fig. S3A). Our previous chain mechanism model of flagella growth proposed that captured subunits remained linked within the export channel with a head-to-tail linkage of N- and C-termini, forming a long subunit chain from the export machinery to the distal flagella tip, where they subsequently assemble into the nascent structure (17). Alternative models for flagellar growth have been proposed, such as subunit diffusion-based models (24–26). Renault et al. demonstrated that deletions within the N-terminal and C-terminal regions of the late export subunit flagellin (FliC) do not affect the rate of flagella growth and proposed an injection-diffusion mechanism whereby subunits are “injected” into the export channel after which they diffuse to the flagella tip and assemble into the nascent structure (24). These results, along with the results in this study, are consistent with the view that specific sequences within subunit termini are not required for subunit capture into a multi-subunit chain.

A previous study showed that early flagellar subunits exert a dominant-negative effect on export and cell motility when overexpressed in wild-type *Salmonella* (FlgD and FliK were not tested in this study) (10). Here we show that deletion of the FlgE C-terminus suppresses this expression phenotype, indicating that the C-terminal export signal causes this previously observed dominant-negative phenotype (Fig. 1B and C). Export of two other early subunits, FlgC and FlgG, was attenuated when their C-termini were deleted, indicating that these subunits also contain a C-terminal export signal (Fig. 2A through D). Unlike FlgD and FliK, all other early subunits are thought to have a C-terminal D_0_ domain which could contain a C-terminal export signal, like those in FlgC, FlgE, and FlgG (27) (Fig. S5).

To test whether the hook cap subunit FlgD also contains a C-terminal export signal, we performed export assays with FlgD variants either containing or lacking their C-termini. Surprisingly, FlgD∆Ct was exported into culture supernatants at the same level as wild-type FlgD (Fig. 2C). Furthermore, neither FlgD∆Ct nor wild-type FlgD displayed a dominant-negative phenotype when overexpressed, indicating that FlgD does not contain a C-terminal export signal (Fig. S3B). Structures of FlgD show that the C-terminus do not contain a D_0_ domain with the C-terminal deletion, resulting in the deletion of four beta sheets and a small alpha helix deletion of which is likely to cause a considerable structural perturbation such that FlgD is unlikely to interact with other FlgD copies and assemble into a pentameric cap (Fig. 3C). This may explain the observation that FlgD∆Ct variants were unable to capture FlgD subunits from FlhB_C_ in *in vitro* capture assays in Evans et al., 2013 (17).

The data indicate that the C-terminal export signal is a subunit targeting signal to aid early subunit entry into the export pathway. In some species, dedicated export chaperones have been identified for the injectisome needle subunit, SctF—a subunit that assembles to form the injectisome needle—a substructure equivalent to the flagellar hook (28, 29). Deletion of the SctF chaperone attenuates needle subunit export; furthermore, this chaperone binds the C-terminus of the needle subunit and, presumably, like other T3SS chaperones, could deliver SctF to SctV (injectisome homolog of FlhA) (12, 19). Unlike SctF, the early flagellar subunits do not have dedicated export chaperones. However, the late flagellar subunits are chaperoned to the export machinery (30–32). The chaperones for the late flagellar subunits also bind the subunit C-terminus and deliver them to various components of the export machinery (11, 12, 19). Deletion of flagellar export chaperones can be suppressed by overexpressing the cognate subunit(s) of the deleted chaperone, indicating that loss of chaperone-mediated targeting can be suppressed by increasing the amount of available subunit in the cell, analogous to suppression of the FlgE∆Ct export defect by overexpression (20).

We provide evidence indicating that the FlgE C-terminus does not dock at the C-ring or ATPase complex components of the export machinery. Overexpression of wild-type FlgE attenuated subunit export to a greater amount than FlgE∆Ct in strains deleted for the C-ring and/or the ATPase, indicating that the binding site of the FlgE C-terminus is not the C-ring or ATPase complex but may instead be one of the core components of the export machinery: FlhA or FlhB.

To identify regions of the FlgE C-terminus that are responsible for the dominant-negative overexpression phenotype, we performed motility assays with a *recA* null strain overexpressing FlgE variants containing 10-residue scanning deletions (Fig. 4A and B). We found that amino acids 371–380 of FlgE were required to give the dominant-negative phenotype of FlgE. Further refinement showed that amino acids isoleucine-376 and arginine-380 are the key residues involved (Fig. 5). A screen for suppressors in cells expressing FlgE I376A or FlgE R380A idenureified six suppressor mutations, all of which increased FlgE expression, indicating that increasing the amount of FlgE in the cell increases the probability of FlgE entry into the export pathway (Fig. S6C).

Interestingly, FlhAc can adopt an open or closed conformation, whereby the closed conformation reduces the binding affinity of FlhAc for the export chaperones (33–35). Formation of the open conformation is required for chaperone binding and efficient late subunit export (33). This indicates that the closed FlhAc conformation may be adopted during early subunit export. It is feasible that this closed FlhAc conformation allows the C-terminus of early subunits to bind FlhAc or to another component of the export machinery, and following the export specificity switch, the adoption of the open FlhAc conformation may prevent early subunits from being efficiently targeted to the export machinery (33).

The data indicate that early flagellar subunits interact sequentially with the export machinery with export signals carrying out distinct roles for subunit targeting, docking at the export machinery, and triggering opening of the export gate. These findings have important implications in understanding the mechanism of assembly of the flagellar type III secretion system. Subunits must be efficiently targeted to the type III export machinery to facilitate rapid protein export (estimated at 1,700 amino acids per second at early stages of export (24). Identifying and understanding the export signals, the sequence in which they are used and their interactions with the export machinery allow us to better understand this complex transport process and have implications for the design of novel anti-infectives targeted against type III secretion systems. In summary, we have presented evidence that the C-termini of early flagellar subunits do not capture subunits from FlhB_C_ as predicted by the chain mechanism model but instead function as a targeting export signal to aid subunit delivery into the export pathway. Refinement of the C-terminal signal identified key residues required for efficient subunit export and motility. We propose that the C-terminal signal aids early subunit targeting to the export machinery by providing a binding site for a core component of the flagellar type III secretion machinery.

## MATERIALS AND METHODS

### Bacterial strains, plasmids, and growth conditions

Wild-type *Salmonella enterica* serovar Typhimurium SJW1103 is motile (36). SJW1103 derived strains (key resources table) were constructed using the λ red recombinase system (37, 38). Resistance cassettes were removed where appropriate using the aph-I-SceI kanamycin resistance cassette replacement using pWRG730 (37). The *Salmonella recA* null strain was used for overexpression studies as the *recA* null strain prevents recombination between the plasmid-borne subunit variants and the chromosomal copy of the gene of interest, which would interfere with interpretation of these assays (). The *Salmonella* Δ*recA* strain is wild type for flagellar protein export and motility. Bacteria were cultured at 30°C–37°C in Luria-Bertani (LB) broth. Recombinant proteins were expressed in *Salmonella* from the IPTG-inducible plasmid pTrc99a (39). To construct recombinant plasmids encoding wild-type or derivative genes, *Salmonella* genes were amplified from chromosomal DNA by PCR or overlap-extension PCR using Q5 High-Fidelity DNA polymerase. PCR products were inserted into pTrc99a using NdeI/HindIII or NdeI/BamHI. Inserts were verified by DNA sequencing (Department of Biochemistry, University of Cambridge). A full list and description of strains and plasmids used in this study can be found in Table 2.

**TABLE 2:** Plasmids, vectors, and reagents used in this study

Reagent type (species) or resource	Designation	Source or reference	Identifiers	Additional information
Strain, strain background (*Salmonella enterica* serovar Typhimurium)	SJW1103	(30)	Wild type	This strain can be obtained from the Fraser lab upon request.
Strain, strain background (*Salmonella enterica* serovar Typhimurium)	*recA* null	(7)	*recA* gene replaced with kanamycin resistance cassette	This strain can be obtained from the Fraser lab upon request.
Strain, strain background (*Salmonella enterica* serovar Typhimurium)	*flgD* null	(17)	*flgD* gene replaced with kanamycin resistance cassette	This strain can be obtained from the Fraser lab upon request.
Strain, strain background (*Salmonella enterica* serovar Typhimurium)	*flgE* null	This work	*flgE* gene replaced with kanamycin resistance cassette	This strain can be obtained from the Fraser lab upon request.
Strain, strain background (*Salmonella enterica* serovar Typhimurium)	*flhB P28T, fliHI null, flgM null*	(14)	P28T mutation introduced into chromosomal *flhB* gene, *flgHI* genes removed by scarless mutagenesis, *flgM* gene replaced by spectinomycin resistance cassette	This strain can be obtained from the Fraser lab upon request.
Strain, strain background (*Salmonella enterica* serovar Typhimurium)	*flhB P28T*, *fliHI* null, *flgM* null, *fliMN* null	This work	P28T mutation introduced into chromosomal *flhB* gene, *flgHI* and *fliMN* genes removed by scarless mutagenesis, *flgM* gene replaced by spectinomycin resistance cassette	This strain can be obtained from the Fraser lab upon request.
Strain, strain background (*Salmonella enterica* serovar Typhimurium)	*flgE* 234(3FLAG)235	This work	Triple flag tag inserted between residue 234 and 234 of *flgE gene*	This strain can be obtained from the Fraser lab upon request.
Recombinant DNA reagent	pTrc99a FlgE	(7)	1–234, FLAG × 3, 235–403 aa	This vector can be obtained from the Fraser lab upon request.
Recombinant DNA reagent	pTrc99a FlgE∆Ct	This work	1–234, FLAG × 3, 235–359 aa	This vector can be obtained from the Fraser lab upon request.
Recombinant DNA reagent	pTrc99a FlgG	(7)	1–144, FLAG × 3, 145–260 aa	This vector can be obtained from the Fraser lab upon request.
Recombinant DNA reagent	pTrc99a FlgG∆Ct	This work	1–144, FLAG × 3, 145–217 aa	This vector can be obtained from the Fraser lab upon request.
Recombinant DNA reagent	pTrc99a FlgC	This work	1–69, FLAG × 3, 70–134 aa	This vector can be obtained from the Fraser lab upon request.
Recombinant DNA reagent	pTrc99a FlgC∆Ct	This work	1–69, FLAG × 3, 70–90 aa	This vector can be obtained from the Fraser lab upon request.
Recombinant DNA reagent	pTrc99a FlgD	This work	1–172, FLAG × 3, 173–232 aa	This vector can be obtained from the Fraser lab upon request.
Recombinant DNA reagent	pTrc99a FlgD∆Ct	This work	1–172, FLAG × 3, 173–190 aa	This vector can be obtained from the Fraser lab upon request.
Recombinant DNA reagent	pTrc99a FlgE∆341–350	This work	1–234, FLAG × 3, 235–340, 351–403 aa	This vector can be obtained from the Fraser lab upon request.
Recombinant DNA reagent	pTrc99a FlgE∆351–360	This work	1–234, FLAG × 3, 235–360, 361–403 aa	This vector can be obtained from the Fraser lab upon request.
Recombinant DNA reagent	pTrc99a FlgE∆361–370	This work	1–234, FLAG × 3, 235–360, 371–403 aa	This vector can be obtained from the Fraser lab upon request.
Recombinant DNA reagent	pTrc99a FlgE∆371–380	This work	1–234, FLAG × 3, 235–370, 381–403 aa	This vector can be obtained from the Fraser lab upon request.
Recombinant DNA reagent	pTrc99a FlgE∆381–390	This work	1–234, FLAG × 3 , 235–380, 391–403 aa	This vector can be obtained from the Fraser lab upon request.
Recombinant DNA reagent	pTrc99a FlgE∆391–403	This work	1–234, FLAG × 3, 235–390 aa	This vector can be obtained from the Fraser lab upon request.
Recombinant DNA reagent	pTrc99a FlgE V_366_A	This work	1–234, FLAG × 3, 235–403 aa, V366A	This vector can be obtained from the Fraser lab upon request.
Recombinant DNA reagent	pTrc99a FlgE E_371_A	This work	1–234, FLAG × 3, 235–403 aa, E371A	This vector can be obtained from the Fraser lab upon request.
Recombinant DNA reagent	pTrc99a FlgE L_372_A	This work	1–234, FLAG × 3, 235–403 aa, L372A	This vector can be obtained from the Fraser lab upon request.
Recombinant DNA reagent	pTrc99a FlgE V_373_A	This work	1–234, FLAG × 3, 235–403 aa, V373A	This vector can be obtained from the Fraser lab upon request.
Recombinant DNA reagent	pTrc99a FlgE I_376_A	This work	1–234, FLAG × 3, 235–403 aa, I376A	This vector can be obtained from the Fraser lab upon request.
Recombinant DNA reagent	pTrc99a FlgE V_377_A	This work	1–234, FLAG × 3, 235–403 aa, V377A	This vector can be obtained from the Fraser lab upon request.
Recombinant DNA reagent	pTrc99a FlgE Q_379_A	This work	1–234, FLAG × 3, 235–403 aa, Q379A	This vector can be obtained from the Fraser lab upon request.
Recombinant DNA reagent	pTrc99a FlgE R_380_A	This work	1–234, FLAG × 3, 235–403 aa, R380A	This vector can be obtained from the Fraser lab upon request.
Recombinant DNA reagent	pTrc99a FlgE Y_382_A	This work	1–234, FLAGx3, 235–403 aa, Y382A	This vector can be obtained from the Fraser lab upon request.
Recombinant DNA reagent	pTrc99a FlgEshort + linker	(7)	1–8, 4× (Gly-Ser-Thr-Asn-Ala-Ser), 33–234, FLAG × 3, 235–403 aa	This vector can be obtained from the Fraser lab upon request.
Recombinant DNA reagent	pTrc99a FlgEshort	(7)	1–8, 33–234, FLAG × 3, 235–403 aa	This vector can be obtained from the Fraser lab upon request.
Recombinant DNA reagent	pTrc99a FlgE∆GRM	This work	1–39, 44–234, FLAG × 3, 235–403 aa	This vector can be obtained from the Fraser lab upon request.
Antibody	Anti-FLAG (mouse monoclonal)	Sigma-Aldrich	Cat # F3165, RRID:AB_259529	Mouse monoclonal against FLAG tag (1:1,000)
Antibody	Anti-FlgD (rabbit polyclonal)	(17)		Rabbit polyclonal against *Salmonella* FlgD (1:1,000) This antibody can be obtained from the Fraser lab upon request.
Antibody	Anti-FliC (rabbit polyclonal)	(17)		Rabbit polyclonal against *Salmonella* FliC (1:1,000). This antibody can be obtained from the Fraser lab upon request.
Antibody	Anti-FlgL (rabbit polyclonal)	(7)		Rabbit polyclonal against *Salmonella* FlgL (1:1,000). This antibody can be obtained from the Fraser lab upon request.
Antibody	Anti-FlgN (rabbit polyclonal)	(15)		Rabbit polyclonal against *Salmonella* FlgN (1:1,000). This antibody can be obtained from the Fraser lab upon request.
Antibody	Anti-FlhA (rabbit polyclonal)	(15)		Rabbit polyclonal against *Salmonella* FlhA (1:1,000). This antibody can be obtained from the Fraser lab upon request.
Antibody	Anti-GroEL (rabbit polyclonal)	Sigma-Aldrich	Cat # G6532	Rabbit polyclonal against FLAG tag (1:40,000)

### Flagellar subunit export assay

*Salmonella* strains were cultured at 37°C in LB broth containing 100-µg/mL ampicillin and 50, 100-µM or 1-mM IPTG to mid-log phase (*A*_600 nm_ 0.6–0.8). Accumulated exported proteins were removed from initial cultures by pelleting the cells, by centrifuging (6,000 × *g*, 5 min) the cells, and by resuspending the cells in fresh media (replacing the media in this way means that the media does not contain any exported proteins at *t* = 0 min and allows us to monitor how protein was exported into the culture media over an hour, at *t* = 60 min). Cells were grown for a further 60 min at 37°C. Cells were pelleted by centrifugation (16,000 × *g*, 5 min), and the supernatant passed through a 0.2-µm nitrocellulose filter. Exported proteins in supernatants were precipitated with 10% trichloroacetic acid and 1% Triton-X100 on ice for 1 h, pelleted by centrifugation (16,000 × *g*, 10 min), washed with ice-cold acetone and resuspended in SDS-PAGE loading buffer [volumes calibrated according to cell densities (*A*_600 nm_)]. Fractions were analyzed by immunoblotting.

### Motility assays

*Salmonella* strains were grown in LB broth to an *A*_600 nm_ of 1. Two microliters of culture was inoculated into soft-tryptone agar (0.25% agar, 10-g/L tryptone, 5-g/L NaCl). Plates were incubated at 37°C for between 4 and 6 h. Agar was supplemented with ampicillin (100 µg/mL) and IPTG where appropriate.

### Isolation of motile strains carrying suppressor mutations

Cells of the *Salmonella* ∆*flgE* strain transformed with pTrc99a plasmids expressing FlgE variants (FlgE I376A or FlgE R380A) were cultured at 37°C in LB broth containing ampicillin (100 µg/mL) and IPTG (50 µM) to mid-log phase and inoculated into soft-tryptone agar (0.3% agar, 10-g/L tryptone, 5-g/L NaCl) containing ampicillin (100 µg/mL) and IPTG (50 µM). Plates were incubated at 37°C until motile “spurs” appeared. Cells from the spurs were streaked to a single colony and cultured to isolate the *flgE* encoding plasmid. Plasmids were transformed into the *Salmonella flgE* null strain to assess whether the plasmids were responsible for the motile suppressor phenotypes. Plasmids were sequenced to identify the suppressor mutations.

### Quantification and statistical analysis

Experiments were performed at least three times. Immunoblot data were quantified using Image Studio Lite. Unpaired two-tailed Student *t*-test was used to determine *P* values, and significance was defined as *P < 0.05. Data are represented as mean ± standard error of the mean, unless otherwise specified and reported as biological replicates.

## SUPPLEMENTAL MATERIAL

Fig. S1 mbio.03067-23-s0001.tiff Motility and expression phenotypes of wild-type FlgE and variants.

Fig. S2 mbio.03067-23-s0002.tiff Effect on subunit export by expression of FlgEshort and variants in a non-assembling strain.

Fig. S3 mbio.03067-23-s0003.tiff Export and motility phenotypes of FlgD and FlgE subunits.

Fig. S4 mbio.03067-23-s0004.tiff Motility phenotype of a *Salmonella* strain deleted for genes encoding flagellar ATPase components and suppressor mutants.

Fig. S5 mbio.03067-23-s0005.tiff Amino acid sequence conservation within the C-termini of early flagellar subunits.

Fig. S6 mbio.03067-23-s0006.tiff Expression levels of wild-type FlgE and variants.

Fig. S7 mbio.03067-23-s0007.tiff Effect on subunit export by overexpression of FlgEshort and variants in a non-assembling strain.

ASM does not own the copyrights to Supplemental Material that may be linked to, or accessed through, an article. The authors have granted ASM a non-exclusive, world-wide license to publish the Supplemental Material files. Please contact the corresponding author directly for reuse.
