# Supplementary figures and images for: Identification of a new export signal that targets early subunits to the flagellar type III secretion export machinery
Source: mBio. 2024 Feb 20;15(3):e03067-23. doi: 10.1128/mbio.03067-23 (PMC10936197; doi:10.1128/mbio.03067-23)

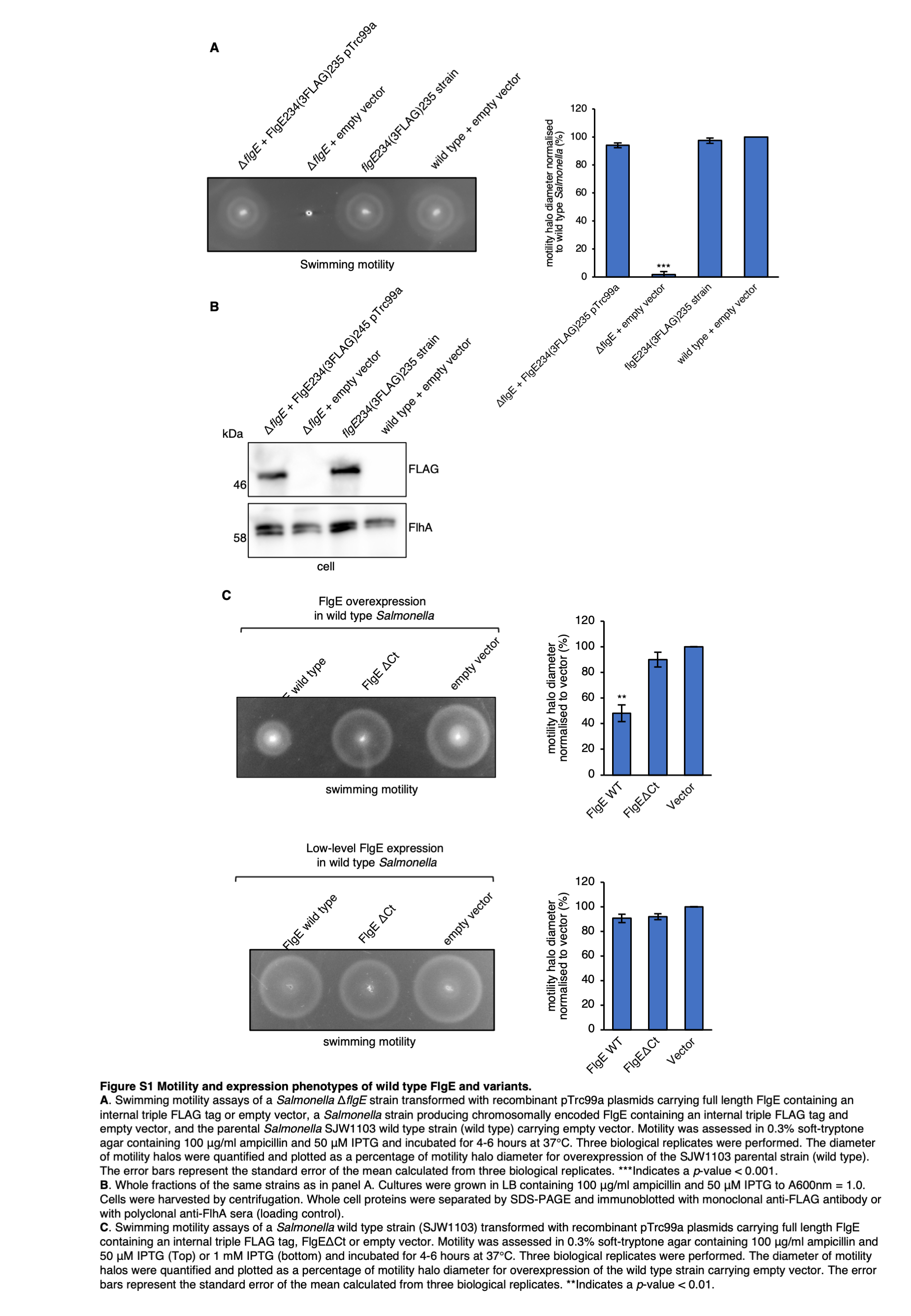

Supplement: Fig. S1 — Motility and expression phenotypes of wild-type FlgE and variants. [file mbio.03067-23-s0001.tiff]

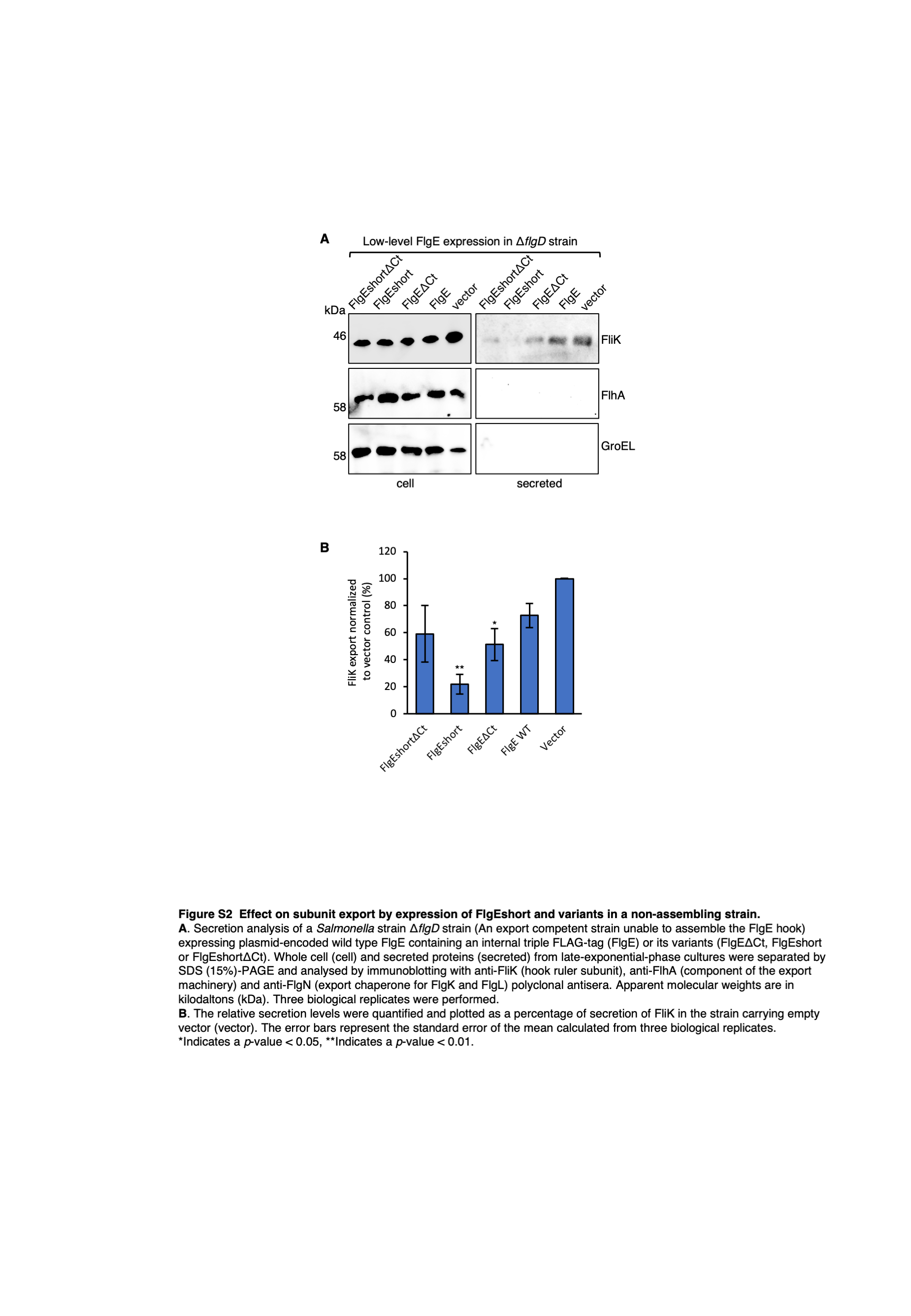

Supplement: Fig. S2 — Effect on subunit export by expression of FlgEshort and variants in a non-assembling strain. [file mbio.03067-23-s0002.tiff]

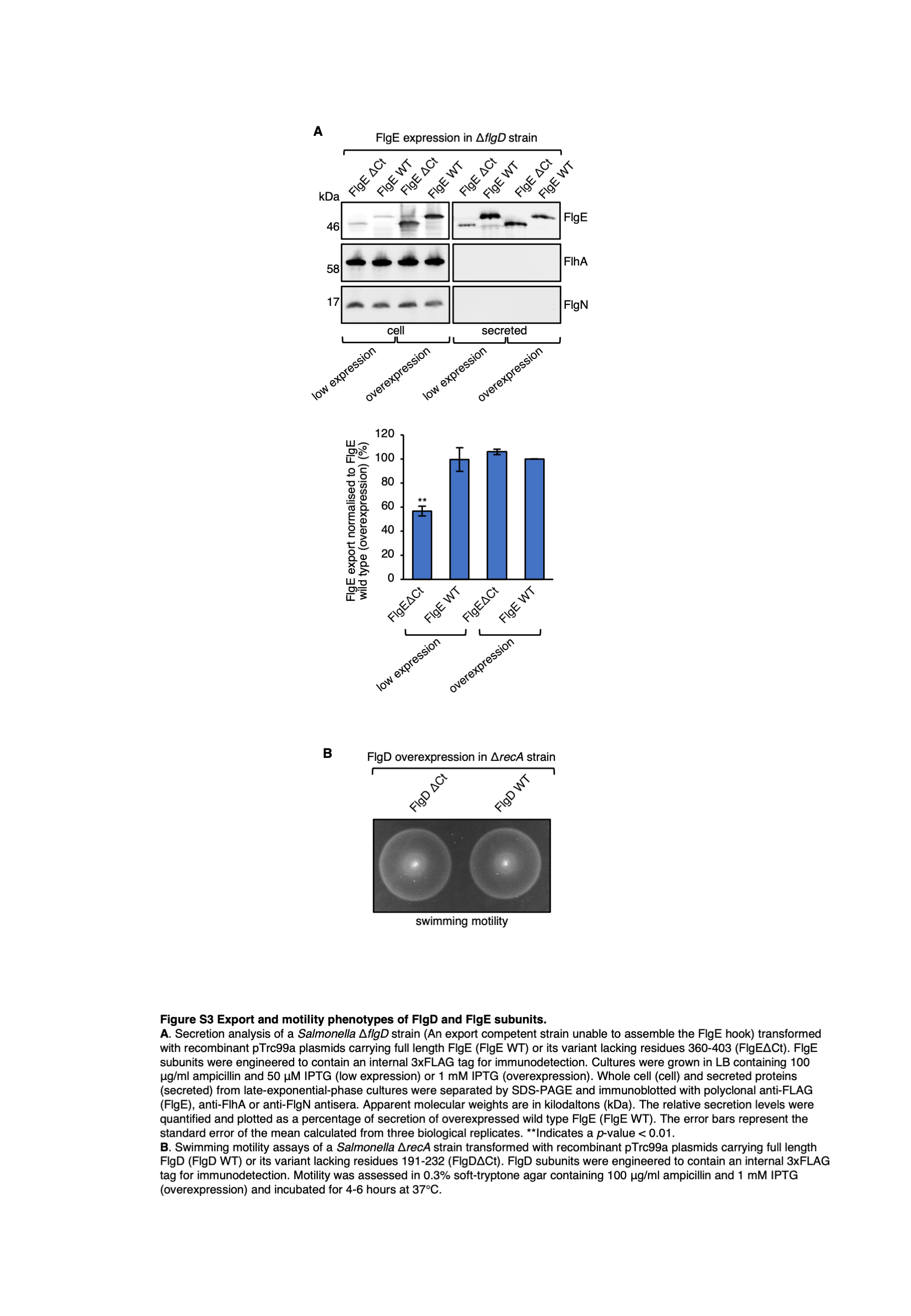

Supplement: Fig. S3 — Export and motility phenotypes of FlgD and FlgE subunits. [file mbio.03067-23-s0003.tiff]

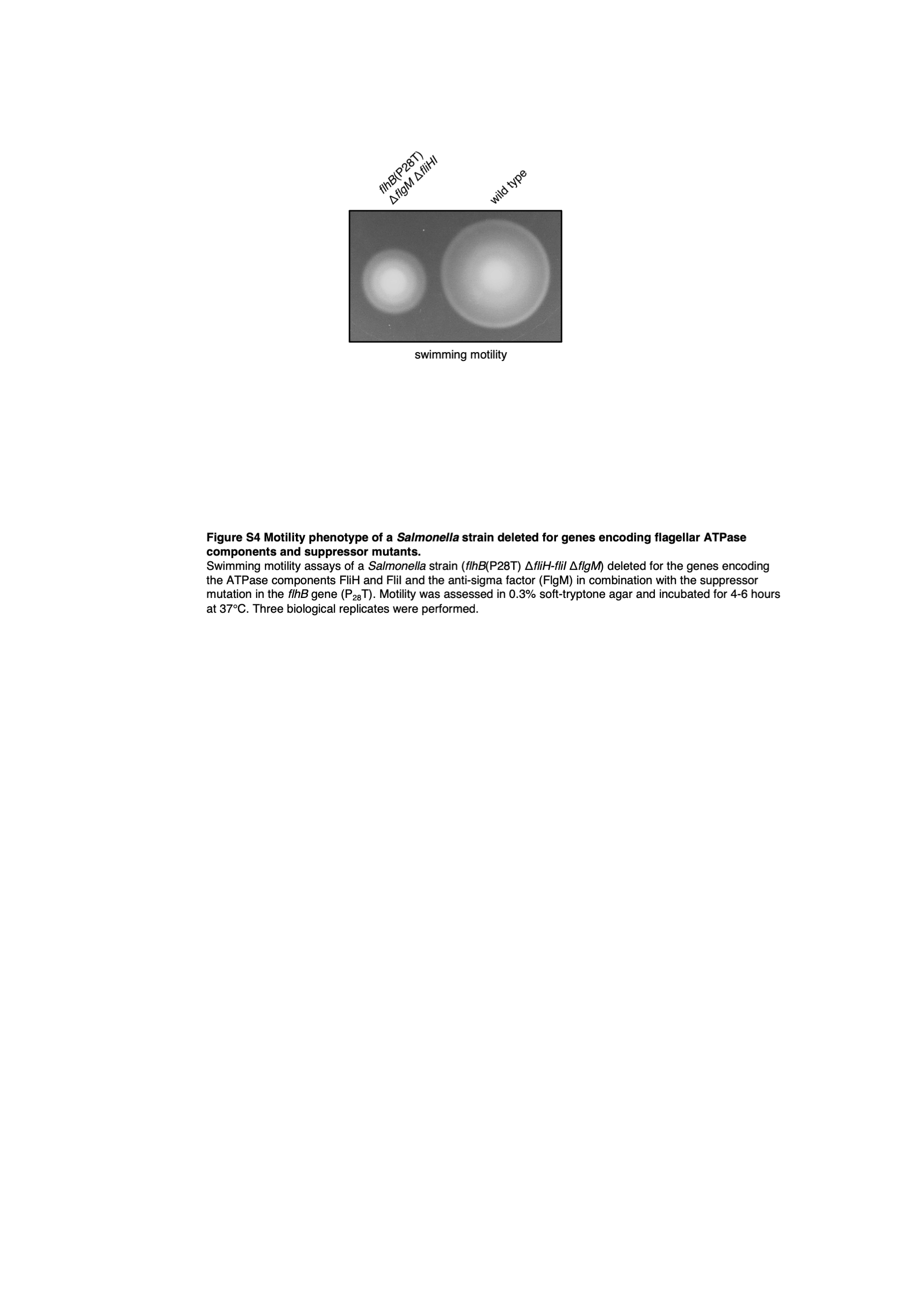

Supplement: Fig. S4 — Motility phenotype of a Salmonella strain deleted for genes encoding flagellar ATPase components and suppressor mutants. [file mbio.03067-23-s0004.tiff]

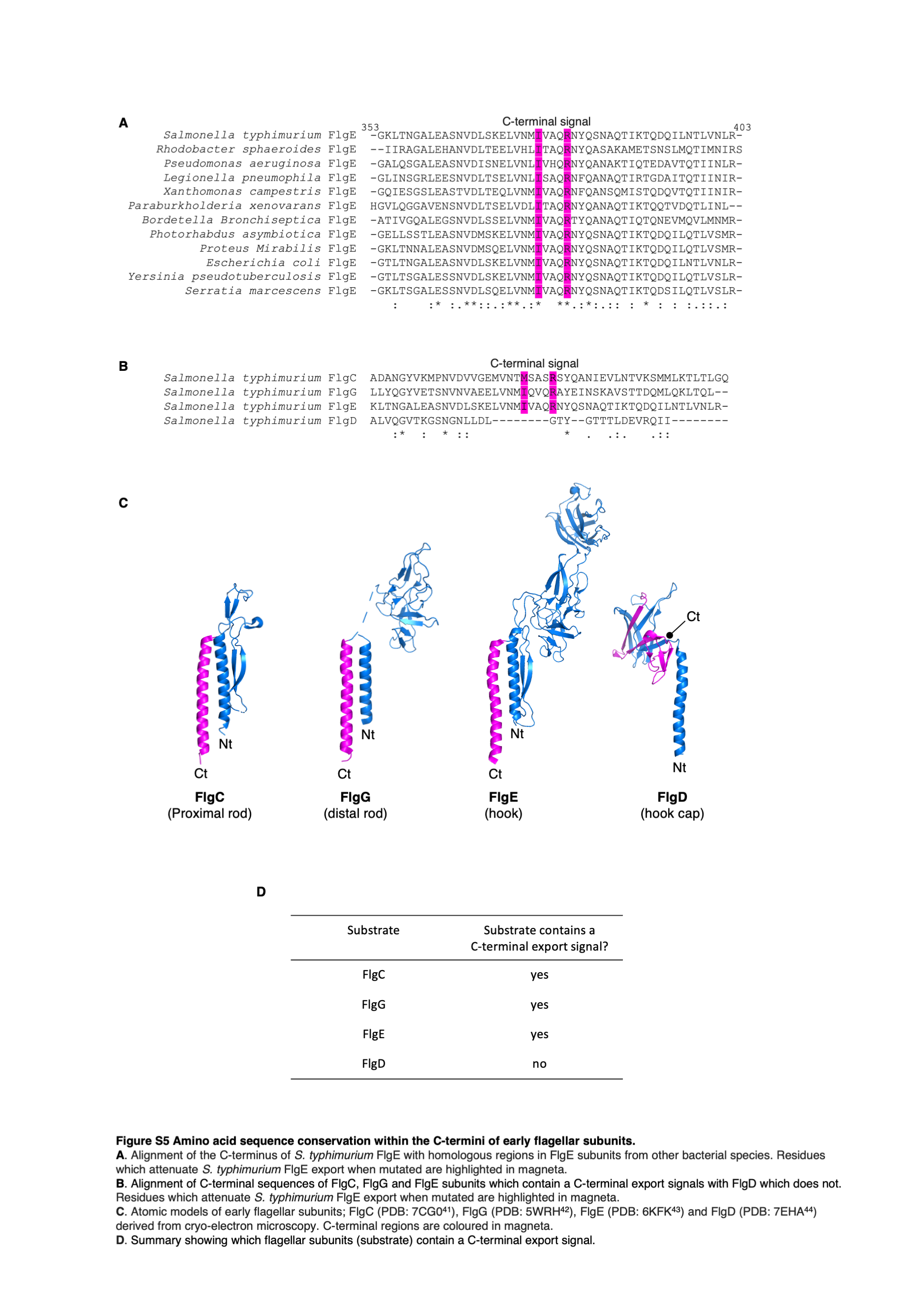

Supplement: Fig. S5 — Amino acid sequence conservation within the C-termini of early flagellar subunits. [file mbio.03067-23-s0005.tiff]

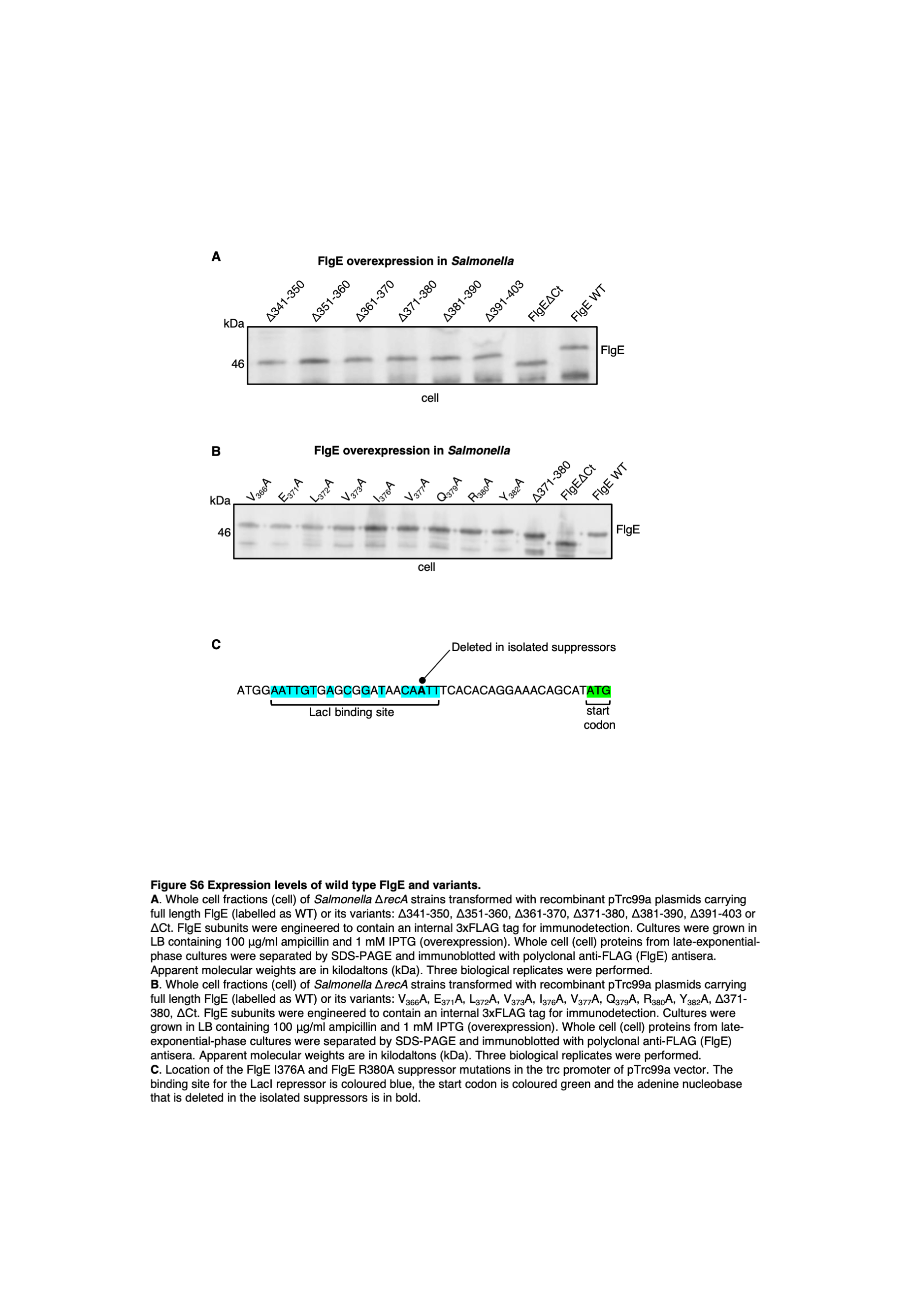

Supplement: Fig. S6 — Expression levels of wild-type FlgE and variants. [file mbio.03067-23-s0006.tiff]

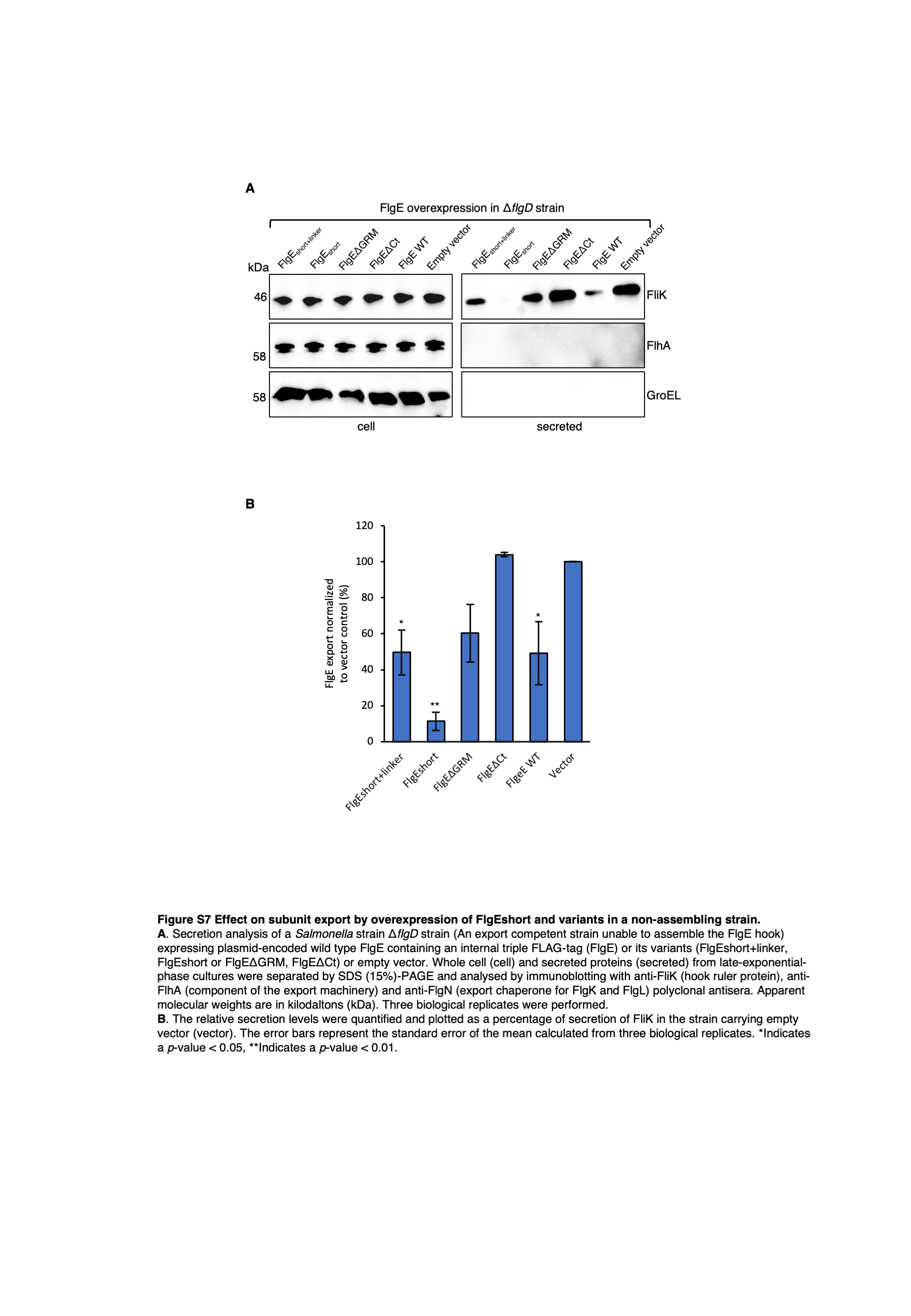

Supplement: Fig. S7 — Effect on subunit export by overexpression of FlgEshort and variants in a non-assembling strain. [file mbio.03067-23-s0007.tiff]
